# CSF Neurofilament Light Chain Elevation Predicts ALS Severity and Progression

**DOI:** 10.3389/fneur.2020.00919

**Published:** 2020-08-28

**Authors:** Qionghua Sun, Xue Zhao, Siyuan Li, Fei Yang, Hongfen Wang, Fang Cui, Xusheng Huang

**Affiliations:** ^1^Neurological Department of the First Medical Center, Chinese PLA General Hospital, Beijing, China; ^2^College of Medicine, Nankai University, Tianjin, China; ^3^Neurological Department of Hainan Hospital, Chinese PLA General Hospital, Sanya, China

**Keywords:** amyotrophic lateral sclerosis, neurofilament light chain, NFL, CSF, axonal damage

## Abstract

**Objectives:** This study compared neurofilament light chain (NFL) levels in the cerebrospinal fluid (CSF) of patients with sporadic amyotrophic lateral sclerosis (sALS) with levels in patients with other neurological diseases and healthy controls and assessed correlations between NFL levels and clinical indicators of sALS.

**Methods:** We used enzyme-linked immunosorbent assays to determine the NFL levels in the CSF of 45 patients with sALS, 21 patients with other central nervous system diseases (OCNSDs), 18 with immune-mediated peripheral neuropathy (IMPN), 14 with non-immune-mediated peripheral neuropathy (NIMPN), and 19 healthy controls (HCs).

**Results:** The median NFL levels in the CSF of the sALS, OCNSD, IMPN, NIMPN, and HC groups were 6510, 5372, 4320, 1477, and 756 pg/mL, respectively. The CSF NFL levels did not differ significantly among the sALS, IMPN, and OCNSD groups, but were significantly higher than those of the NIMPN and HC groups. The NFL CSF levels were significantly higher in the NIMPN group than the HCs. There was a negative correlation between the NFL level and ALS function score (ALSFRS-R), and a positive correlation with the disease progression rate in patients with sALS.

**Conclusion:** CSF NFL may not be sufficient to distinguish ALS from other central nervous system diseases or peripheral neuropathy, but it predicts ALS severity and progression.

## Introduction

Amyotrophic lateral sclerosis (ALS) is a fatal neurodegenerative disease that selectively involves the upper and lower motor neurons. The pathogenesis of ALS remains unclear, but axonal injury may be involved (1). The diagnosis of ALS is based mainly on clinical features, electromyography, and the exclusion of other diseases. Many recent studies have focused on the role of neurofilaments (NFs) as biomarkers in ALS (2). NFs are cytoskeletal proteins and their levels increase in biological fluids proportionally to the degree of axon damage. NFs consist of light (NFL), medium (NFM), and heavy (NFH) chain subunits. There is growing evidence that the levels of NFs, especially phosphorylated neurofilament heavy chains (p-NFH) and neurofilament light chains (NFL), reflect axonal injury and are potentially of value in ALS and other neurological disorders with axon damage. Cerebrospinal fluid (CSF) and blood levels of NFs are increased in ALS (3–13) and other neurological disorders, including Alzheimer's disease (14, 15), multiple sclerosis (16, 17), multiple system atrophy predominated by parkinsonism (18), Charcot–Marie–Tooth disease (19), and cerebrovascular disease (20). NFs are non-specific markers of axon damage. To determine whether NFL can distinguish ALS from other nervous system diseases, we determined the NFL levels in CSF from patients with sALS and compared the levels in other nervous system diseases. Furthermore, some studies have observed that NFL levels were correlated with the clinical upper motor neuron burden (7, 21, 22), and increased levels were associated with a poor prognosis of ALS (23–27). Therefore, we also assessed the relationship between NFL levels and clinical indicators of sALS.

## Materials and Methods

### Patients

The study enrolled 45 patients diagnosed with sALS admitted to the Department of Neurology at the Chinese PLA General Hospital from December 2017 to December 2018. The diagnosis was based on the Revised E1 Escorial diagnostic criteria (28). Three disease control groups were also enrolled: 20 patients with other central nervous system diseases (OCNSDs) [seven cases of multiple system atrophy (MSA), four of subacute combined degeneration (SCD), two of multiple sclerosis (MS), and one case each of corticobasal degeneration, spasmodic torticollis, progressive supranuclear palsy, spinocerebellar ataxia, Alzheimer's disease, adrenoleukodystrophy, and acute myelitis]; 18 patients with immune-mediated peripheral neuropathy (IMPN) [five with chronic inflammatory demyelinating polyradiculoneuropathy (CIDP), three with Guillain–Barré syndrome (GBS), one with chronic idiopathic axonal polyneuropathy, and nine with other immune-mediated peripheral neuropathies]; and 14 patients with non-immune-mediated peripheral neuropathy (NIMPN), such as metabolic and hereditary peripheral neuropathy. Another 19 patients with primary migraine and somatization disorder were enrolled as healthy controls (HCs). Participants were excluded from the study if they had brain trauma, cerebral infarction, or other conditions that alter CSF NFL levels (29).

### Clinical Parameters of ALS and Sample Collection

The clinical data of the 45 patients with sALS were recorded, including age, gender, site of symptom onset, disease duration (time from symptom onset to sample collection), diagnostic category (clinically definite, clinically probable, clinically probable-laboratory-supported, or clinically possible ALS), and the time of symptom spread from spinal or bulbar localization to generalization (TTG), which is an early clinical parameter of disease progression (30), as well as clinical phenotypes, including upper (UMND-ALS) and lower (LMND-ALS) motor neuron dominant ALS or classical ALS, according to the extent of UMN and LMN involvement and areas affected at the time of sampling (31). The onset site was classified as bulbar or limb onset. We also determined the score on the ALS functional rating scale revised (ALSFRS-r) (32). The disease progression rate (DPR) was calculated as [(48 − ALSFRS-r score at evaluation)/(disease duration from symptom onset to evaluation in months)] (33). The forced vital capacity (FVC) of the ALS patients was also tested.

CSF samples were collected from all patients and controls at the time of diagnosis in our department after lumbar puncture for CSF analysis. All samples were centrifuged at 3,000 rpm at 4°C for 10 min within 2 h of collection. The supernatants were stored at −80°C until tested. This study was approved by the ethics committee of the Chinese PLA General Hospital. Informed consent was obtained from all participants.

## NFL ELISA

The NFL CSF concentrations were measured using commercial enzyme-linked immunosorbent assays (ELISAs; IBL International, Hamburg, Germany), performed in accordance with the manufacturer's instructions. The range 100–10,000 ng/L was explored. Samples were run in duplicate, together with freshly prepared standards, and positive and negative controls on each plate.

### Statistical Analysis

For continuous variables, the Kolmogorov–Smirnov test was used to determine whether the data were normally distributed and Levene's test to determine the homogeneity of the variance. Normally distributed data are expressed as the mean ± standard deviation and non-normally distributed data as the median and interquartile range (IQR). The CSF NFL levels were non-normally distributed. Differences in the CSF NFL levels between the HCs and sALS or OCNSD groups were determined by analysis of covariance adjusted for age. Wilcoxon's non-parametric test was used to compare the NFL levels among the other groups. Spearman correlation analysis was used to analyze the associations between the CSF NFL level and clinical parameters in the sALS patients. The analyses were performed using SPSS (ver. 20.0; SPSS, Chicago, IL, USA). The level of statistical significance was set at *p* < 0.05.

## Results

### Demographic Characteristics of the Subjects

Table 1 shows the age and gender of all subjects. The NFL concentration in all subjects was significantly correlated with age (*r* = 0.339, *p* < 0.0001), but not with gender (*r* = 0.060, *p* = 0.518). Among the five groups, significant differences were found in age between the HCs and sALS (*p* = 0.016) and OCNSD (*p* = 0.010) groups. To avoid age-related bias when comparing NFL levels, differences in CSF NFL levels between the HCs and sALS and OCNSD groups were analyzed using analysis of covariance after adjusting for age (Table 1).

**Table 1:** Demographic characteristics of the study groups.

**Group**	***n***	**Male/Female (n)**	**Mean age, years (SD)**
sALS	45	25/20	54.29 (8.81)
OCNSD	20	16/4	56.75 (13.76)
IMPN	18	12/6	52.06 (15.43)
NIMPN	14	10/4	45.21 (14.13)
HC	19	13/6	40.11 (16.28)

*ALS, amyotrophic lateral sclerosis; OCNSD, other central nervous system diseases; IMPN, immune-mediated peripheral neuropathy; NIMPN, non-immune-mediated peripheral neuropathy; HC, healthy control; SD, standard deviation*.

### Clinical Parameters of the ALS Group

The study enrolled 45 patients with sALS (25 males, 20 females). Of these, 28 were diagnosed with clinically definite ALS, 15 with clinically probable ALS, and two with clinically possible ALS. Eight cases were classified as bulbar onset and 37 as limb onset ALS. The mean age at sampling was 54.29 ± 8.81 years. The median disease duration was 12 (range 3–84) months. There were 37 patients with classical ALS and four each with UMND-ALS and LMND-ALS. The mean ALSFRS-r score was 37.29 ± 8.54 and the mean DPR was 0.94 ± 0.85. The mean FVC%, which was obtained from 39 ALS patients, was 87.23 ± 21.17%. Twenty-two cases progressed to generalized ALS and the mean TTG was 12.18 ± 11.30 months.

### Comparison of NFL Levels Between ALS Patients and Controls

**Figure 1:**
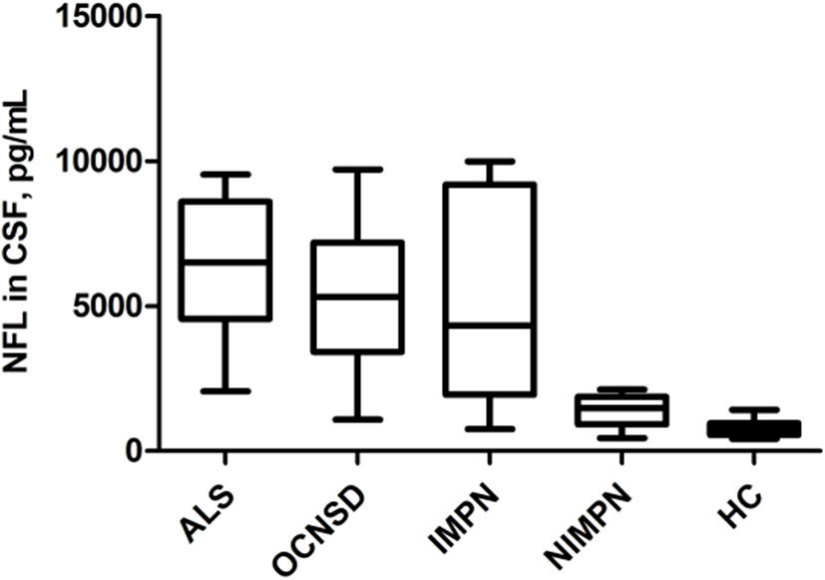
Comparison of NFL levels in CSF of different groups.

The median CSF NFL levels in the sALS, OCNSD, IMPN, NIMPN, and HC groups were 6510, 5307, 4320, 1477, and 756 pg/mL, respectively (Table 1). No significant differences were observed in NFL levels among the sALS, OCNSD, and IMPN groups. The CSF NFL levels were significantly higher in these three groups than in the NIMPN and HC groups. The CSF NFL level was significantly higher in the NIMPN group than in the HC group (Table 2 and Figure 1).

**Table 2:** NFL levels in CSF of patients from different groups.

***p-value***	**NFL(pg/ml)**	**sALS**	**OCNSD**	**IMPN**	**NIMPN**	**HC**
sALS	6510 (4557–8598)	/	0.091	0.132	<0.0001	<0.0001
OCNSD	5307 (3413–7181)	0.091	/	0.520	<0.0001	<0.0001
IMPN	4320 (1948–9183)	0.132	0.520	/	0.001	<0.0001
NIMPN	1477 (913–1875)	<0.0001	<0.0001	0.001	/	0.001
HC	756 (551–960)	<0.0001	<0.0001	<0.0001	0.001	/

CSF, cerebrospinal fluid; NFL, neurofilament light chain; ALS, amyotrophic lateral sclerosis; OCNSD, other central nervous system diseases; IMPN, immune-mediated peripheral neuropathy; NIMPN, non-immune-mediated peripheral neuropathy; HC, healthy control.

*NFL levels are expressed as median values (interquartile range). The p-value in bold was < 0.05, indicating that the NFL levels of the corresponding two groups were statistically different*.

### Associations Between NFL Levels and ALS Clinical Parameters

**Figure 2:**
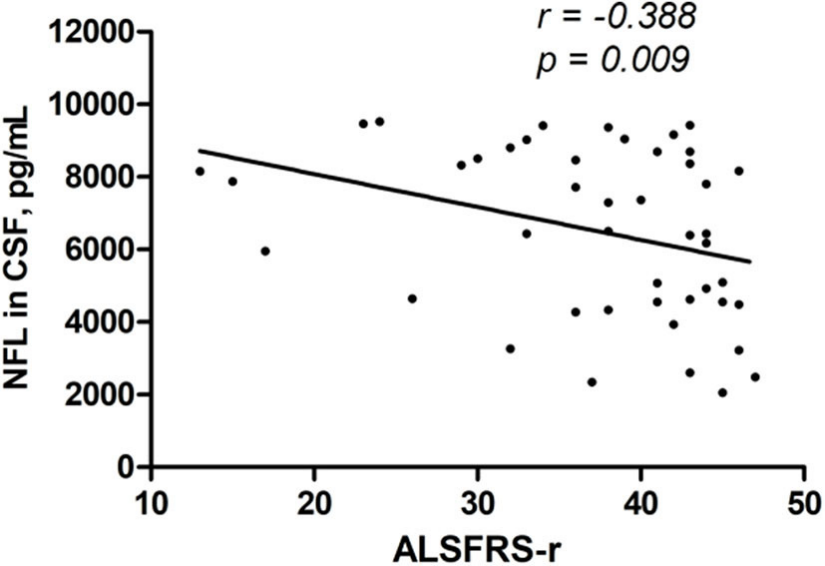
CSF NFL level was negatively correlated with the ALSFRS-r (*r* = − 0.388, *p* = 0.009).

**Figure 3:**
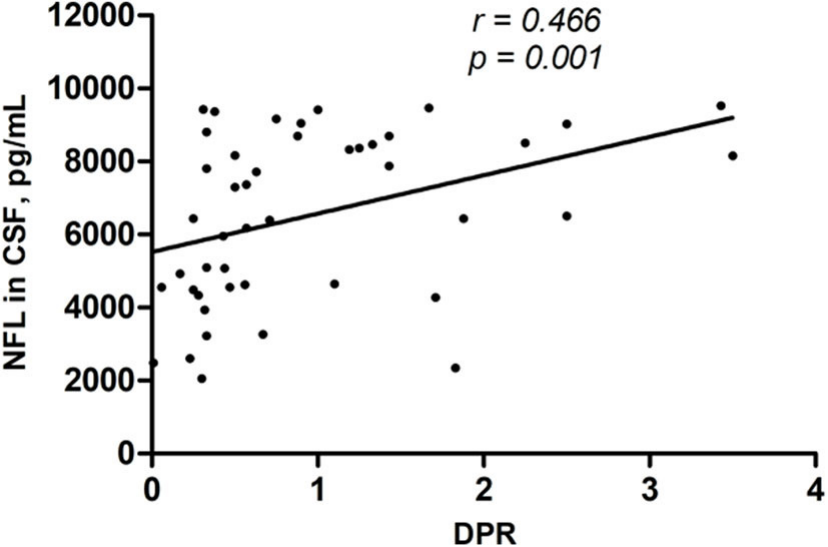
CSF NFL level was positively correlated with DPR (*r* = 0.466, *p* = 0.001).

Using Spearman correlation analysis, there were no significant correlations between NFL level and age at onset, onset site, diagnostic category, clinical phenotype, TTG, or FVC. A significant negative correlation was found between NFL level and ALSFRS-r and a significant positive correlation between NFL level and DPR (Table 3 and Figures 2, 3).

**Table 3:** Correlations between the levels of NFL and ALS clinical parameters.

**Clinical parameters**	***R***	***p-value***
Age at onset	−0.133	0.383
Site of onset	−0.201	0.185
Disease duration	−0.190	0.212
Diagnostic category	−0.060	0.695
Clinical phenotype	−0.050	0.743
TTG	−0.339	0.123
FVC	−0.154	0.350
**ALSFRS-r**	**−0.388**	**0.009**
**DPR**	**0.466**	**0.001**

ALS, amyotrophic lateral sclerosis; NFL, neurofilament light chain; Disease duration, time from symptom onset to sample collection; ALSFRS-r, revised ALS functional rating scale; DPR, disease progression rate; FVC, forced vital capacity; TTG, the time of symptoms spreading from spinal or bulbar localization to generalization.

The values represent the correlations between NFL level and each clinical parameter.

*Correlations were considered significant at p < 0.05. The p < 0.05 was in bold*.

## Discussion

In this prospective study, the CSF NFL levels were significantly higher in patients with sALS than healthy controls, but were not significantly elevated compared with patients with OCNSD or IMPN, which are expected to lead to significant axon damage. We confirmed that NFL levels are promising prognostic biomarkers for monitoring disease severity and disease progression of sALS.

In recent years, there has been strong evidence from independent groups, large cohorts of patients, and multicenter studies that neurofilament light chains are potentially valuable ALS biomarkers (3, 5–13, 21, 23). Verde et al. showed that serum NFL is increased in ALS compared to other conditions and can serve as a diagnostic and prognostic biomarker using ultrasensitive single-molecule array technology (6). Gaiani et al. confirmed the role of NFL as a diagnostic and prognostic biomarker in ALS, and suggested that elevated CSF NFL levels in patients with upper motor neuron involvement and FTD reflect corticospinal tract degeneration, while low NFL levels in patients with lower motor neuron signs indicate milder disease phenotypes (7). In a multicenter study, Feneberg et al. showed that CSF and serum NF concentrations discriminate ALS with early symptom onset from other neurological diseases (9). In a relatively large cohort of 190 ALS patients and 130 with other neurological diseases, Rossi et al. confirmed that CSF NFL and p-NFH were valuable diagnostic and prognostic biomarkers in ALS (10). A prospective, multicenter, longitudinal study validated NfL values as prognostic biomarker for ALS, comparing serum and CSF NfL values (13). Tortelli et al. found that CSF NFL levels were increased in ALS compared to other neurodegenerative disorders and peripheral neuropathies and were correlated with disease severity and progression (23). Contrasting these studies, we found that CSF NFL levels were significantly elevated not only in sALS patients but also in the OCNSD and IMPN groups compared to healthy controls. Comparing ALS, OCNSD, and IMPN patients, the ability of CSF NFL to discriminate groups was low and did not differ significantly. It seems CSF NFLs are non-specific markers present in various neurological diseases that cause significant axon damage. Considering the small number of cases include of our study compared to previous research, a large sample size may be more convincing. Increased CSF NFL levels in patients with CIDP, GBS, or IMPN may be due to the release of axonal protein after nerve root injury (34). Elevated CSF NFL levels in OCNSDs, such as MSA, SCD, and MS, may reflect different degrees of pyramidal tract damage. Furthermore, we observed that the CSF NFL levels in NIMPN were higher than that in HCs and lower than in the IMPN group. We speculated that the NFL concentration in CSF released after nerve root injury is higher than that induced by peripheral nerve injury.

We found a positive correlation between CSF NFL levels and the rate of disease progression and a significant negative correlation between CSF NFL levels and ALS function scores. The rate of disease progression based on the ALS function score is an important indicator of the clinical progression and deterioration of ALS patients (33). Our work is in line with reports that CSF NFL performs relatively well as a prognostic biomarker for ALS. In our ALS sample, we did not find a significant correlation between the NFL concentration and TTG, another disease progression indicator, while another study found a short TTG in patients with high NFL levels (3). This is likely because only 22 of our patients have progressed to spinal and bulbar involvement and have complete TTG data; a small sample size cannot reflect the relationship between the CSF NFL concentration and TTG well. Some studies have also suggested that the CSF NFL levels are higher in ALS patients with predominantly upper motor neuron involvement (7, 22), which we did not observe. Of the 45 sALS patients in our study, only four had UMND-ALS. The relatively small proportion may be one of the causes of the phenomenon. In addition, the extent of pyramidal tract damage in classic ALS may not be less than that of prevalent upper motor neuron ALS.

There were some shortcomings to our work. First, the sample size is relatively small; a large sample size may be more convincing. Second, we assayed only the CSF NFL concentration, and not that in blood. A comparison of blood and CSF NFL levels in patients may better explain the results. In addition, serum measurements are more favorable to monitor disease progression, and the reliability of NFL assay also in serum was widely reported in literature. Third, all patients in our study underwent therapies (Riluzole or Edaravone) at the time of lumbar puncture. We did not record the detailed treatment process, which possibly influenced axonal preservation or damage, and future study should consider it.

In conclusion, the CSF NFL levels are increased in patients with sALS and other nervous system diseases. The high CSF NFL levels of sALS patients were associated with more rapidly evolving disease and more severe disability due to ALS.

## Data Availability Statement

The raw data supporting the conclusions of this article will be made available by the authors, without undue reservation.

## Ethics Statement

The studies involving human participants were reviewed and approved by the ethics committee of the Chinese PLA General Hospital. The patients/participants provided their written informed consent to participate in this study.

## Author Contributions

QS was responsible for the design of the subject, the implementation of the experiment, and the writing of the article. XZ was responsible for the collection of sample. SL was responsible for the guidance of the experiment. FY, HW, and FC were responsible for the guidance of the subject. XH was in charge of the project, responsible for the project's funding, design, and checking of the article.

## Conflict of Interest

The authors declare that the research was conducted in the absence of any commercial or financial relationships that could be construed as a potential conflict of interest.
